# Management of Vestibular Bone Fenestration with Periosteal Inhibition (PI) Technique During Alveolar Socket Preservation: A Case Report

**DOI:** 10.3390/medicina60121912

**Published:** 2024-11-21

**Authors:** Andrea Grassi, Maria Eleonora Bizzoca, Lucia De Biasi, Rossella Padula, Ciro Annicchiarico, Gabriele Cervino, Lorenzo Lo Muzio, Filiberto Mastrangelo

**Affiliations:** 1 Independent Researcher, 42121 Reggio Emilia, Italy; grassi@dentistire.it; 2 Department of Clinical and Experimental Medicine, University of Foggia, 71122 Foggia, Italy; mariaeleonora.bizzoca@unifg.it (M.E.B.); lucia_debiasi.564354@unifg.it (L.D.B.); lorenzo.lomuzio@unifg.it (L.L.M.); 3 Independent Researcher, 70124 Bari, Italy; annicchiarico.ciro63@gmail.com; 4 Morphological and Functional Images, Department of Biomedical and Dental Sciences, University of Messina, 98100 Messina, Italy; gabriele.cervino@unime.it

**Keywords:** bone defect, vestibular fenestration, periosteal inhibition, PI technique, cortical lamina, blood clot, Degidi’s chamber, alveolar ridge preservation, dental implant, implant survival

## Abstract

*Background and Objectives:* The purpose of this case report is to examine the management of vestibular bone fenestration during alveolar socket preservation using the Periosteal Inhibition (PI) approach. Here, for the first time, the PI technique, which has been shown to be successful in maintaining intact cortical bone, is examined in the context of a bone defect. *Materials and Methods*: After an atraumatic extraction of a damaged tooth, a vestibular bone fenestration was discovered in the 62-year-old male patient. To shield the defect, a non-resorbable PTFE membrane (OSSEO GUARD by Zimmer Biomet) was positioned between the mucosa and the fenestration site. A resorbable porcine gelatin sponge (SPONGOSTAN^TM^) was used to achieve hemostasis, and a 5/0 PGCL absorbable suture was used to close the wound. A CBCT scan was performed, and a dental implant was inserted after 4 months. *Results*: After 4 months, the case demonstrated positive results, with full cortical remodeling and preservation of the original bone proportions. The fenestration completely healed, proving that the PI approach works even in the presence of bone flaws in cortical bone that is still intact. *Conclusions*: This is the first case report that shows that vestibular bone fenestration can be successfully treated with the PI approach. It has now been demonstrated that the procedure, which hitherto needed an undamaged cortical bone to work, can help bone abnormalities to repair completely. These results imply that the PI technique is a flexible and useful approach that provides predictable results in dental surgery for treating different types of alveolar bone abnormalities. Its use might be expanded with more study to include bone dehiscence treatment.

## 1. Introduction

The importance of aesthetics has changed; the use of tissue-integrated implants for the prosthetic replacement of missing teeth has become widely accepted and sought after by both patients and medical practitioners [1]. The fundamental principle of these tissue-integrated implants is a biotechnical process known as osseointegration. Even though this concept has been around and explored for a long time, the increasing understanding of its cellular and molecular mechanisms has prompted researchers to delve deeper into the factors that affect the osseointegration process. This has facilitated the acceleration and enhancement of the osseointegration process by taking advantage of the numerous and even the smallest details and occurrences in this natural process [2].

In the esthetic zone, in the case of tooth extraction, the clinician is often confronted with a challenge regarding the optimal decision-making process for providing a solution using dental implants. This is because, after tooth extraction, alveolar bone loss and structural and compositional changes of the covering soft tissues, as well as morphological alterations, can be expected [3,4]. Ideally, the therapeutic plan starts before tooth extraction, and it offers three options: the spontaneous healing of the extraction socket, immediate implant placement, and techniques of alveolar ridge preservation [5]. The decision-making process mainly depends on the chosen timepoint for implant placement and the ability to place a dental implant, the quality and quantity of soft tissue in the region of the extraction socket, the remaining height of the buccal bone plate, and the expected rates of implant survival and success. Based on scientific evidence, three time periods for alveolar ridge preservation are described in the literature: soft-tissue preservation with 6–8 weeks of healing after tooth extraction (for the optimization of the soft tissues), hard- and soft-tissue preservation with 4–6 months of healing after tooth extraction (for the optimization of the hard and soft tissues), and hard-tissue preservation with >6 months of healing after tooth extraction (for the optimization of the hard tissues) [6].

Post-extraction, the edentulous site undergoes morphological and dimensional alterations of the alveolar ridge, attributable to qualitative and quantitative modifications [3,4,7]. Under physiological circumstances, a clot comprising blood cells, serum, and saliva forms. The thrombus occludes the blood vessels, and a fibrin network, which serves as a scaffold for the migration of neutrophil, monocyte, and fibroblast granulocytes, is established within the first day. In the central region, lymphocytes and leukocytes initiate the haemolysis process, gradually replacing the clot with granulation tissue. The studies conducted by Araujo et al. in 2008 documented a decrease in the horizontal ridge dimension in post-extraction sockets, ranging from 2.6 to 4.6 mm [8], while the mean reduction in vertical ridge height was approximately 1.24 mm. The application of biomaterials to graft the alveolar socket and the use of barrier membranes can mitigate the extent of these dimensional changes [4,9,10].

Scientific evidence defines a defect according to its position as either a supra-osseous defect (horizontal), an intraosseous (vertical) defect, or an interradicular defect. As per the classification of Goldman and Cohen in 1958, horizontal defects are located above of the bony crestal margin [11]. Vertical defects have their bases at the apical margin of the alveolar crest; angular defects and craters are distinguished. Angular defects affect a single tooth element, while craters affect two contiguous teeth. Depending on the residual walls, angular defects are divided into defects with one wall, two walls, or three walls [12].

Bone augmentation procedures are the gold standard for the reconstruction of alveolar ridge defects. However, some procedures are challenging and carry a higher risk of postoperative complications.

All techniques of alveolar ridge preservation (ARP) that involve the use of biomaterials to fill the extraction socket have demonstrated results that are not consistently predictable, while the contraction of volume is often diminished [13]. Moreover, the drawbacks associated with the insertion of a biomaterial, such as prolonged healing times and the generation of viable bone, are significantly fewer than those associated with natural healing or the sole use of collagen [8]. The Socket Shield Technique appears to be an ARP technique capable of preserving the internal osteo-gingival structure, due to the conservation of the buccal portion of the root and the periodontal ligament [14]. This procedure has achieved complete preservation at the 10-year mark [14,15]. The Modified Socket Shield technique, which does not involve implant placement but includes concurrent reconstruction of the extraction socket, was introduced by Glocker [16,17]. The technique’s disadvantages include its execution complexity, operator dependency, extended execution time, and the fact that the remaining root occupies a portion of the implant space.

The PI technique has redirected attention to the exterior of the extraction socket, counteracting the osteolytic activity originating from the deep periosteum [18]. Preliminary findings have indicated the near-total preservation of both hard and soft tissue volumes, without the necessity of retaining root fractions within the socket or using a biomaterial. The benefits of this technique include its extreme simplicity and rapid execution, a relatively short return to normalcy in 4 months, and most importantly, the formation of only vital bone within the extraction socket. The constraints of the PI technique involve the subsequent removal of the d-PTFE membrane and the inability to augment alveolar volumes, as it is only able to preserve them. The previous study of Grassi et al. [18] showed evidence that the Periosteal Inhibition (PI) Technique is effective on intact cortical. This study represents the inaugural instance of a case with vestibular fenestration managed using the PI Technique.

When barrier membranes like PTFE are used for bone preservation, the periosteum is especially important for guided tissue regeneration (GTR) and bone repair. As a source of growth factors and progenitor cells, the periosteum plays a major role in stabilizing the surrounding soft tissue and forming new bone. GTR’s ability to promote bone regeneration by blocking non-osteogenic cells and enabling periosteum-derived cells to improve defect healing was initially shown by Dahlin et al. [19]. In experimental models, studies by Kostopoulos et al. [20] and Weng et al. [21] further demonstrated the periosteum’s critical function in bone regeneration, demonstrating that its preservation promotes osteogenesis and supports the stability of newly formed bone. In a canine buccal dehiscence model, recent research by Ma et al., [22] compared periosteal applications to collagen membranes, confirming that the periosteum facilitates bone formation and defect repair more efficiently. In methods such as the Periosteal Inhibition (PI) approach, which uses the periosteum’s osteogenic capability to enable the predicted bone preservation without the need for graft materials, these findings highlight the significance of preserving periosteal integrity.

Presenting and assessing the Periosteal Inhibition (PI) Technique’s efficacy in treating a vestibular bone fenestration during alveolar socket preservation is the main goal of this research. This case study specifically intends to show how the PI approach, used here for the first time in a context with a cortical bone defect, may maintain stable bone dimensions and encourage bone regeneration without the need for biomaterials. The procedure followed, the clinical and radiological results obtained, and the possible benefits of the PI technique over conventional methods are all thoroughly documented in the study.

## 2. Materials and Methods

### 2.1. Study Design

**Figure 1:**
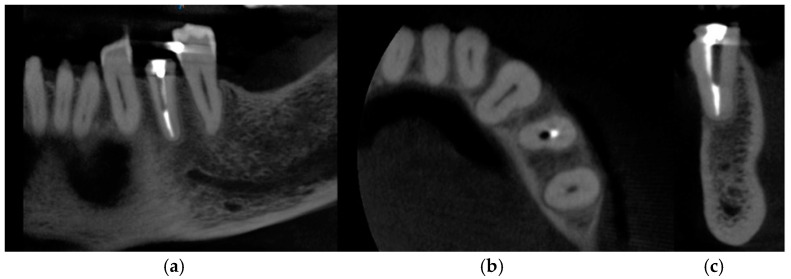
CBCT images of the patient before the surgery: (**a**) frontal section, (**b**) transversal section, and (**c**) sagittal section.

The following case study report was performed in the private clinic Dott. A Grassi, in compliance with the patient who satisfied the inclusion criteria and was included in the present study: male, 62 years old, not a smoker, and in general good health. Written informed consent was signed by the patient for the clinical procedure and for the present study. The patient was provided with a prophylaxis starting the day before the surgery for 6 days, with 2 g of amoxicillin and clavulanic acid. Pre-operative CBCT with a Carestream 8100 3D (Carestream Dental, Atlanta, GA, USA) was performed (Figure 1a–c).

Inclusion Criteria:

- Age > 18 years old;
- General good health (ASA I-II);
- Adequate oral hygiene (Full Mouth Plaque Score ≤ 20%, Full Mouth Bleeding Score ≤ 20%);
- Presence of one or more hopeless teeth requiring extraction.

Exclusion Criteria:

- Pregnancy or lactating period;
- Untreated periodontitis;
- Osteometabolic disease;
- Intravenous bisphosphonates therapy;
- Chemotherapy or radiation therapy history of the neck–head area;
- Heavy smokers (>15 cigarettes/per day);
- Absence of buccal bone plate.

### 2.2. Surgical Protocol

Following local anesthesia utilizing a 4% articaine solution containing 1:200,000 adrenaline, an atraumatic extraction was executed.

Subsequently, an incision of the papillae was made using a #15c surgical scalpel with a full-thickness envelope flap. A fenestration was observed on the buccal bone, the lingual–buccal length was approx. 8.3 mm.

**Figure 2:**
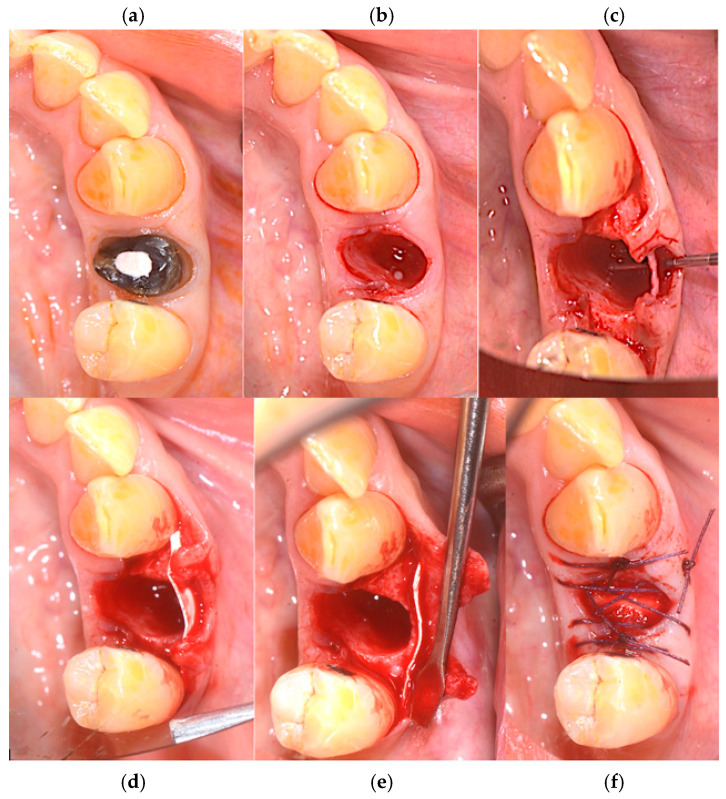
(**a**) Preoperative sites, (**b**) alveolar socket, (**c**) fenestration in the vestibular bone, (**d**,**e**) ptfe OSSEOGUARD membrane from Zimmer Biomet in place, and (**f**) SPONGOSTAN and sling suture.

A ptfe membrane, OSSEOGUARD of Zimmer Biomet (Warsaw, IN, USA), was placed between the mucosa and the fenestration site. To achieve haemostasis, 1 cubic centimeter of SPONGOSTAN™ (Ethicon, Somerville, MA, USA), a sterile gelatin sponge of porcine origin that is malleable, resorbable, and insoluble in water, with a whitish, porous appearance, was inserted into the socket. For suturing, a sling suture was applied to the papillae as well as a central cross, using a 5/0 PGCL absorbable suture from OMNIA (Fidenza, Italy). The sutures were removed after 7–8 days (Figure 2a–f).

**Figure 3:**
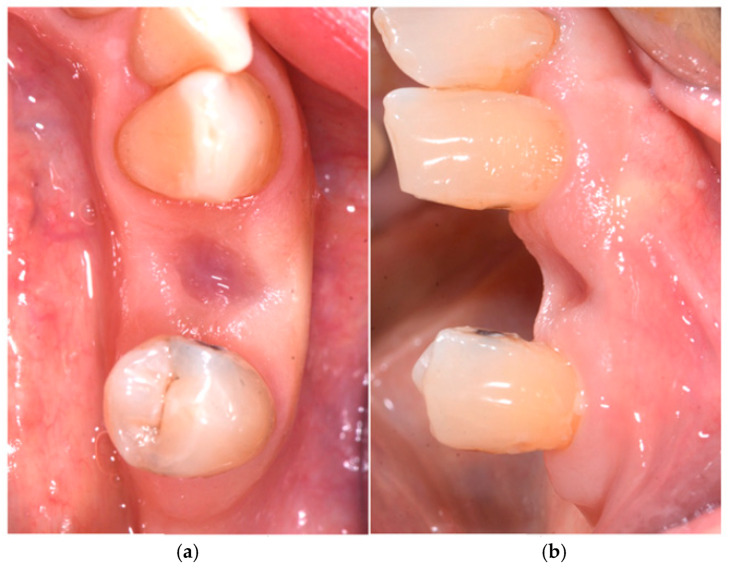
Tissue healing after 1 month: (**a**) occlusal view and (**b**) buccal view.

One month after surgery, a soft-tissue check was performed, which confirmed excellent soft-tissue healing with no loss of volume (Figure 3a,b).

**Figure 4:**
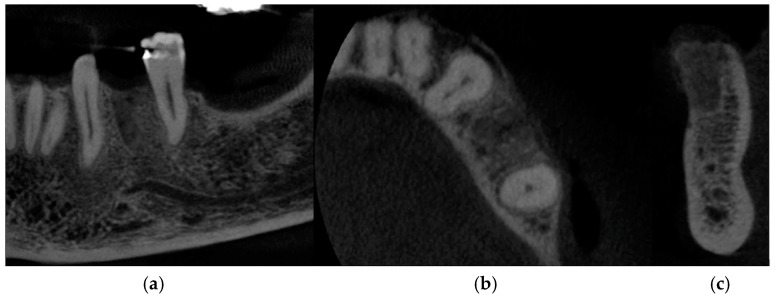
CBCT images at 4 months after the surgery: (**a**) frontal section, (**b**) transversal section, and (**c**) sagittal section.

At 4 months, CBCT was performed to check the hard tissues, which confirmed a correct healing pathway with maintenance of the initial dimensions (Figure 4a–c).

**Figure 5:**
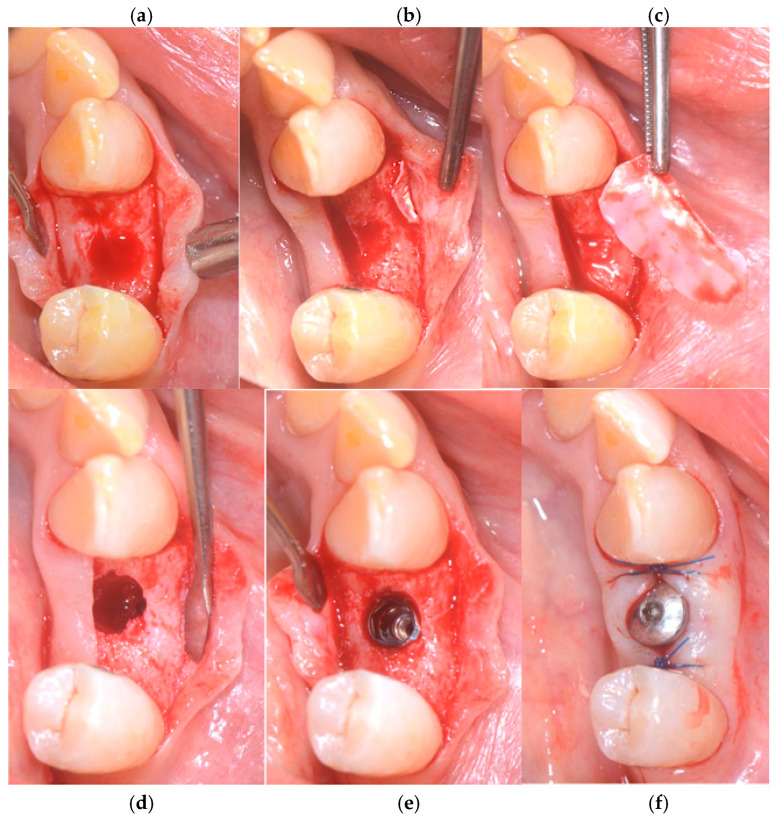
Second surgical step: (**a**) incision for the envelope flap, (**b**) individualizing the membrane, (**c**) removing the membrane, (**d**) site preparation for the implant, (**e**) implant inserted, and (**f**) healing screw in place.

An envelope flap incision was made to remove the ptfe OSSEOGUARD membrane from the site and insert the T3 PRO ZIMVIE IMPLANT (Westminster, CO, USA) with a diameter of 4.1 mm and a length of 10 mm and a healing screw (Figure 5a–f). The implant was placed according to the manufacturer’s instructions provided in the product’s technical sheet.

**Figure 6:**
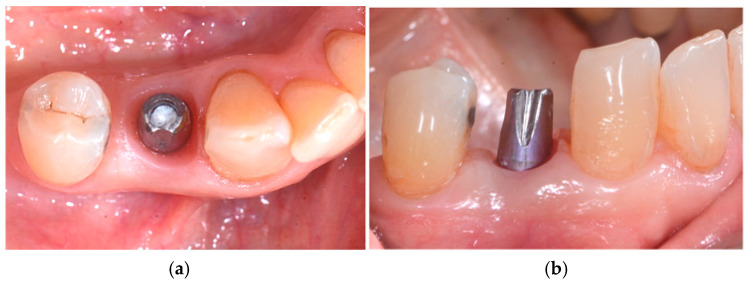
Third surgical step: (**a**) Curvomax abutment occlusal view and (**b**) Curvomax abutment sagittal view. Perfect soft-tissue healing can be observed.

Three months after the placement of the implant, the abutment and the resin provisional prosthetic joint were inserted. The abutment fitted for the prosthesis was a Curvomax from Biomax (Vicenza, Italy) (Figure 6a,b).

**Figure 7:**
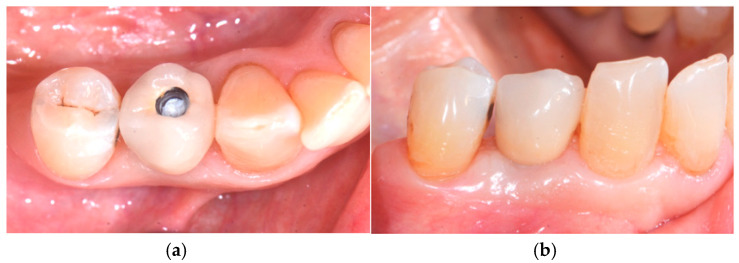
Fourth surgical step: (**a**) prosthetic rehabilitation occlusal view and (**b**) prosthetic rehabilitation—sagittal view.

The final prosthesis was placed 20 days after the provisional prosthetic element (Figure 7a,b). The definitive prosthesis was a screw-retained metal-ceramic model. The connection between the abutment and the crown was achieved using zinc polycarboxylate cement, specifically Popy-F Plus by Dentsply Sirona (Konstanz, Germany).

**Figure 8:**
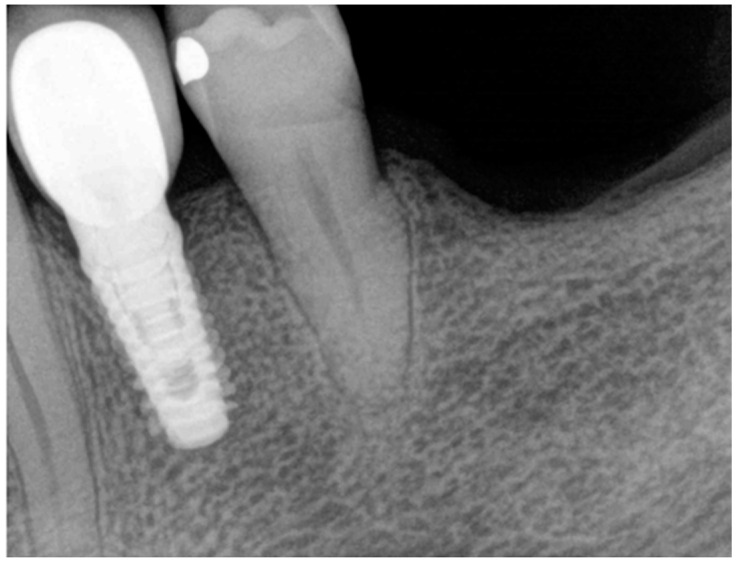
Final endoral RX.

At the end of the work, a control endoral RX was carried out (Figure 8).

## 3. Results

### 3.1. Radiographic Linear Measurements

**Figure 9:**
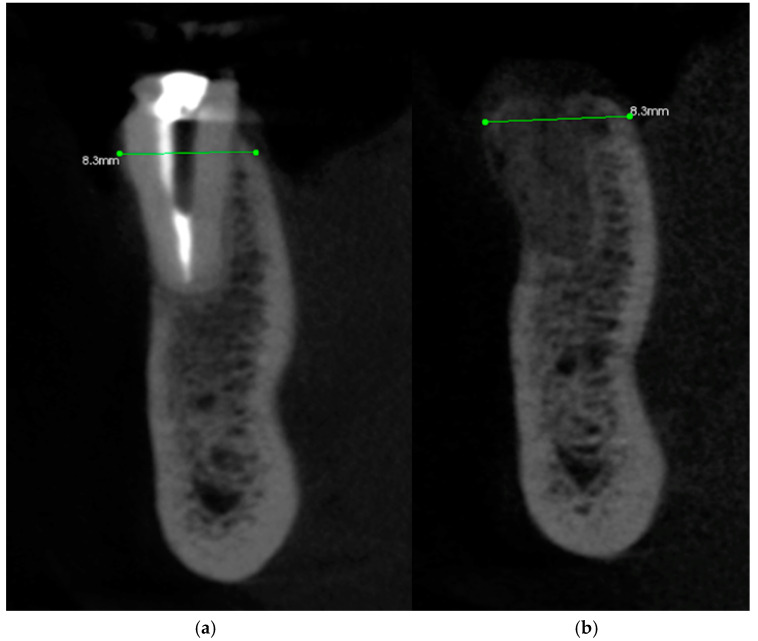
(**a**) CBCT images before the surgery (sagittal section) and (**b**) CBCT images after 4 months (sagittal section).

Taking the remaining teeth as a reference, we positioned ourselves in the same coronal cuts, measuring the bone thickness. At the site of interest, the preoperative CBCT showed a ridge width of 8.30 mm that was maintained (Figure 9a,b). Following a 4-month period, the CBCT follow-up revealed that the alveolar ridge dimensions had been successfully preserved, with the ridge width remaining at 8.30 mm. This result implies that the bone resorption at the fenestration site was successfully inhibited by the Periosteal Inhibition (PI) approach.

### 3.2. Clinical Results

**Figure 10:**
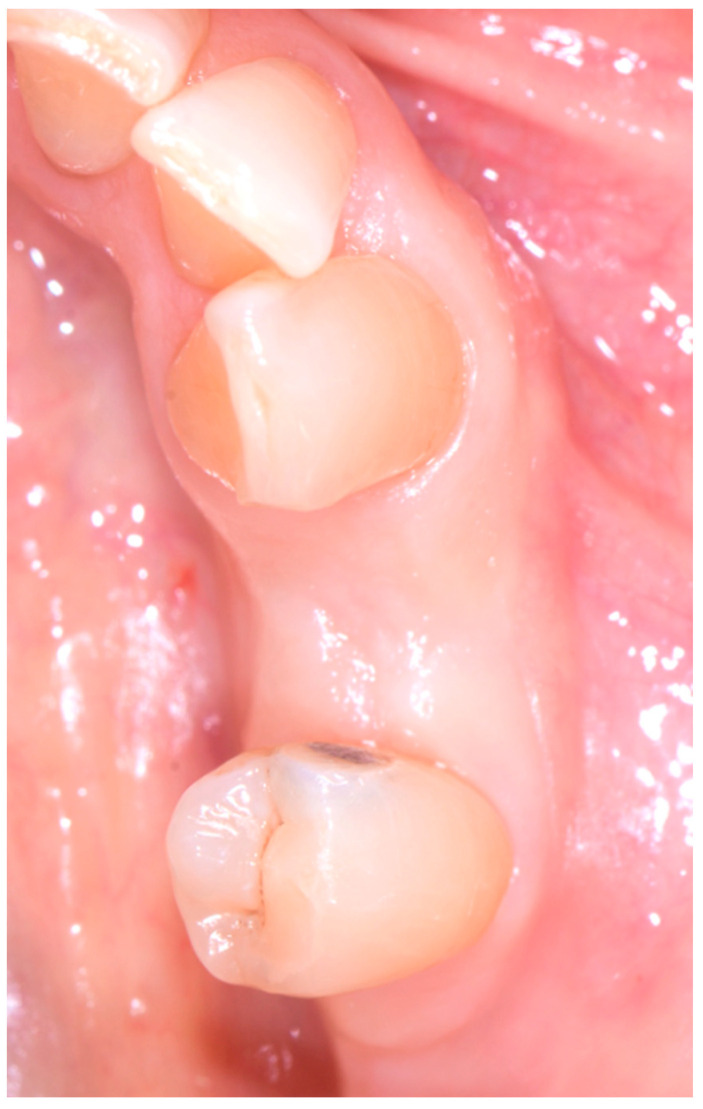
Tissue healing after 4 months (occlusal view).

Soft-tissue recovery was assessed 1 month and 4 months after surgery (Figure 10). The clinical assessment 1 month later revealed remarkable soft-tissue healing without any evidence of volume loss. The tissue seemed completely healed after 4 months, proving that the PI approach was successful in accelerating the recovery of both soft and hard tissues. The lack of side effects, such infection or membrane exposure, adds even more evidence for the technique’s effectiveness and safety.

Overall, this case showed that the PI approach was effective in managing vestibular bone fenestration, maintaining the proportions of the alveolar ridge and encouraging full healing in a span of 4 months. Currently, the follow-up is 8 months, and the results are confirmed.

**Figure 11:**
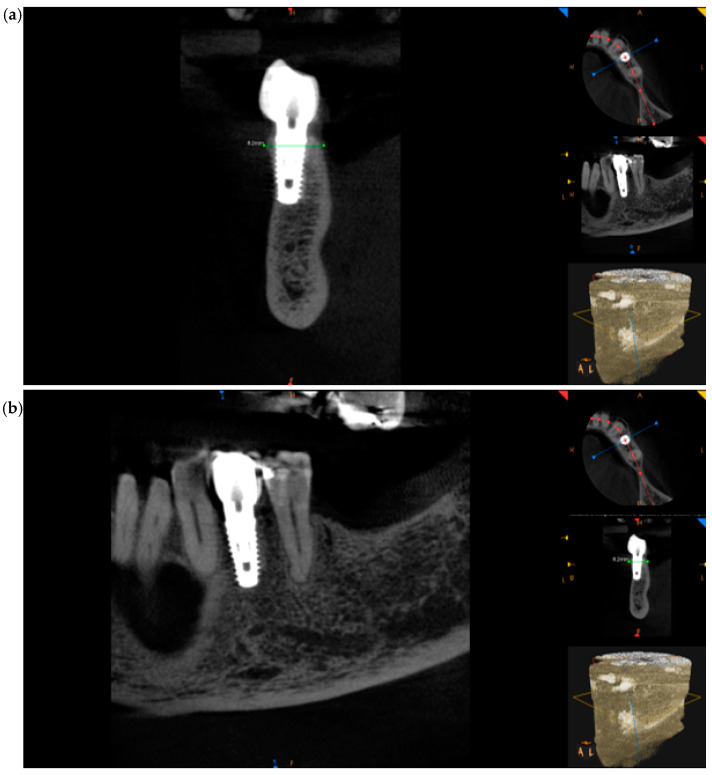
(**a**) CBCT pictures after a year (sagittal section) and (**b**) CBCT pictures after a year (coronal section).

After a year, a cone-beam CT (CBCT) scan was carried out because the patient wanted implant planning in additional locations. This made it possible to evaluate the PI-treated area a year after the prosthetic restoration. The CBCT pictures (Figure 11a,b) show a slight decrease in bone level of just 0.1 mm, suggesting that the initial bone dimensions have been strongly maintained over time. The PI approach may be a dependable choice for situations needing prolonged alveolar ridge preservation, because this additional long-term data supports the evidence for its efficacy in maintaining bone stability.

## 4. Discussion

With its abundance of osteoprogenitor cells and growth factors that promote bone formation, the periosteum is essential for the healing of extraction sockets. By supplying vital cells and biochemical cues to the location, the periosteum aids in the initiation and maintenance of osteogenesis after tooth extraction, fostering the growth of new bone along the alveolar socket. Research has shown that maintaining the periosteal layer, particularly during the early phases of healing, can improve bone healing and preserve the socket’s structural integrity [21,22]. By preventing soft-tissue invasion and focusing osteogenic activity in the defect area, preserving the continuity of the periosteum with the help of the PTFE membrane seems to further maximize healing in the context of the Periosteal Inhibition (PI) approach. This method emphasizes the periosteum’s critical role in the successful preservation of the alveolar ridge by helping to maintain the socket’s vertical and horizontal dimensions while also encouraging the development of strong, vital bone that will be appropriate for the implantation of an implant later.

Alveolar ridge preservation is a fundamental practice in dentistry. This procedure is indicated to safeguard pre-existing hard and soft tissues, maintain a stable bone volume, and simplify subsequent rehabilitation, whether it involves a fixed implant-supported prosthesis or dental prosthesis [16].

Following the extraction of a tooth, dimensional changes occur in the alveolar process, which are inevitable because its morphology is conditioned by the presence of the dental elements themselves. The resorption of the alveolar crest is more marked in the horizontal direction than in the vertical, with a concomitant loss of bone from the vestibular wall. The literature studies have reported a reduction in the horizontal dimensions of the crests in post-extraction sockets from 2.6 to 4.6 mm, while the average vertical reduction in crest height was about 1.24 mm [3,4,7].

The primary objective of alveolar ridge preservation (ARP) upon its initial introduction was to fill the post-extraction socket with bone graft material, followed by coverage with a coronally advanced gingival flap [23]. Subsequently, ARP has been refined with the intent to minimize physiological, vertical, and horizontal bone resorption post-tooth extraction through the combined use of a barrier membrane and bone graft material [24].

For ARP, a variety of graft materials are primarily employed as scaffolds. Autologous bone is considered the gold standard, as it possesses three fundamental properties based on the concept of tissue engineering: osteogenic, osteoinductive, and osteoconductive capabilities, and it does not elicit an antigenic immune response.

However, given the ARP’s requirement for a considerable amount of graft material to fill the post-extraction socket, alternative materials such as allografts, xenografts, and alloplasts are generally used in place of autologous bone, which presents both surgical invasiveness and volume limitation [25,26]. In conclusion, alveolar ridge preservation is an important practice in dentistry, both with the use of graft biomaterials and without. This procedure helps to maintain a stable bone volume and facilitates subsequent prosthetic rehabilitation [16,27].

Modified periosteal inhibition is a surgical technique used to preserve and increase the thickness of the vestibular bone after tooth extraction to counteract the physiological resorption of the bone crest. The technique involves the use of a 0.5 mm thick cortical plate placed between the vestibular periosteum and the cortical bone. The technique involves the use of a membrane placed between the vestibular periosteum and the cortical bone, and the insertion of a 0.5 mm thick cortical plate. The cortical plate is shaped and fixed with human fibrin glue, and a collagen sponge is inserted to stabilize the clot. CBCT exams are performed before the dental extraction and after 4 months to evaluate the effectiveness of the technique. The technique achieves an average increase in vestibular bone thickness of 0.41–0.21 mm, and no early or late complications have been observed [18].

The periosteal inhibition technique presented in 2019 for both alveolar preservation and immediate implant placement shifts the focus from what happens inside the alveolus to what happens outside the cortical bones. The goal of this technique is no longer to fill the space created by dental extraction but to mechanically protect the vestibular cortical bone from the aggression of pre-osteoclasts, which, attracted by the inflammatory–reparative situation, attach to the bone cortex and then form osteoclasts that begin to resorb it [26,28,29]. Initial observations have suggested an almost complete conservation of both hard and soft tissue volumes, eliminating the need to keep root fragments within the socket or employ a biomaterial. The advantages of this method encompass its remarkable simplicity, swift implementation, relatively quick recovery period of 4 months, and crucially, the development of only living bone within the extraction socket [30]. The limitations of the PI method include the subsequent removal of the d-PTFE membrane and its incapacity to enhance alveolar volumes as it can merely preserve them.

This technique was developed for use on intact cortical bone without the need for biomaterials, with the goal of forming only vital bone.

The MPI technique was developed for use on intact cortical bone, fenestration, and little dehiscence (4–5 mm large) without the need for biomaterials, with the goal of forming only vital bone.

One of the main advantages of the PI and MPI techniques is their ability to increase the predictability of the outcome. This is particularly important in a field like dentistry, where precision and the reliability of results are crucial. The PI technique allows for a more accurate prediction of treatment outcomes, thus improving the quality of patient care.

However, one indication of the PI technique was the need for perfect cortical bone. This could limit the applicability of the technique to a narrow range of cases. However, this case demonstrates that the PI technique is effective even in the presence of a fenestration. In fact, the PI technique was used to manage a fenestration, and perfect healing was observed, with the complete repair of the bone defect.

These results broaden the potential applications of the PI technique, making it an even more versatile choice for dental professionals. With its simplicity, effectiveness, and versatility, the PI technique represents a significant advancement in the field of dentistry.

Several clinical findings were made throughout the post-operative period after the vestibular bone fenestration was managed with the Periosteal Inhibition (PI) approach. The instance in question is noteworthy due to its successful healing without any difficulties. This underscores the effectiveness of the PI approach in addressing faults of this nature. Nonetheless, it is important to think about potential issues that can come up in situations like this and comprehend the elements that go into the positive results shown in this particular instance.

It is clear from examining relevant in vivo and clinical research that the Periosteal Inhibition (PI) technique has clear benefits for preserving alveolar ridges, especially when contrasted with more conventional methods like guided bone regeneration (GBR). By using a PTFE membrane to shield the periosteum and cortical bone surface from resorption, the PI technique reduces intervention and preserves bone dimensions using fewer materials and a shorter recovery time than GBR, which usually calls for the use of graft materials and prolonged healing periods [21,22]. Our 12-month follow-up CBCT scan revealed minimal vertical bone loss, demonstrating the effectiveness of this technique in preserving vertical ridge height and sustaining steady bone regeneration over time.

Furthermore, we acknowledge that quantitative assessments like volumetric analysis and bone density would improve the assessment of the effect of the Periosteal Inhibition (PI) approach on bone remodeling. Cone-beam computed tomography (CBCT) and sophisticated imaging software may be used in future research to quantify changes in bone density, volume, and thickness in the defect area. According to research like Cardaropoli et al.’s [3], this method would provide practitioners with quantitative data to more accurately assess clinical success and enable a more accurate evaluation of the PI technique’s long-term results. With the potential to streamline post-extraction treatment and enhance results in clinical situations where long-term ridge stability is crucial, these results establish the PI approach as a practical and effective substitute for socket preservation.

By placing a non-resorbable PTFE membrane on the outside of the extraction socket, the Periosteal Inhibition (PI) approach effectively isolates the periosteum and shields the cortical bone from osteoclastic resorption, helping to preserve the vertical ridge’s dimension. This barrier helps retain the ridge’s vertical dimension by preventing soft-tissue invasion and maintaining an ideal environment for osteogenesis right on the bone surface. This result is corroborated by the extra CBCT scan performed at the 12-month follow-up, which reveals just 0.1 mm of vertical bone loss in comparison to the initial data. According to these results, the PI approach is a good choice for alveolar ridge preservation before implant insertion because it provides efficient long-term ridge height maintenance.

This study’s dependence on a single patient case is one of its main limitations, as it naturally limits how broadly our findings can be applied. Larger-scale research is necessary to confirm the promising early results of the Periosteal Inhibition (PI) approach in treating vestibular bone fenestration across a range of patient populations and therapeutic contexts. Increasing the sample size would enable a more thorough knowledge of how patient-specific characteristics, such as bone density, health status, and healing rates, can affect the efficacy of the PI approach in addition to confirming the reproducibility of our findings. Trombelli et al.’s findings [4] and other earlier research on alveolar ridge preservation have emphasized the necessity of longer-term monitoring and more comprehensive data to evaluate the stability and predictability of bone defect management strategies. Therefore, we support more clinical trials to build a strong evidence base for the PI method, extending its clinical reliability and enabling its use in a larger spectrum of patients.

### 4.1. Potential Complications

The possibility of infection is a major worry after every surgical procedure involving soft tissue and bone [2,7,13]. The strict preoperative and postoperative protocols in this instance, which included the administration of prophylactic antibiotics (amoxicillin and clavulanic acid) and the upkeep of aseptic conditions throughout the procedure, are credited with the lack of infection. In spite of these safeguards, doctors should exercise caution because infections can still occur, particularly in patients with weakened immune systems or poor dental hygiene.

Membrane exposure is a further possible problem. In this instance, the use of a non-resorbable PTFE membrane was essential for preventing the bone defect from repairing and fostering recovery. These membranes may, however, occasionally become visible if the soft tissue above does not heal correctly or is put under too much strain when the sutures are placed. Exposure to a contaminated membrane may need early membrane removal, which may hinder the healing process. In this instance, the effective prevention of membrane exposure was probably made possible by the cautious use of a sling suture and the non-traumatic manipulation of the tissues.

Another problem that might have happened but was not seen in this instance is soft-tissue dehiscence. Inadequate flap design, too much suture tension, or early suture removal can all lead to dehiscence [2,7,13]. The lack of soft-tissue dehiscence in this patient indicates that the surgical approach, which included using absorbable sutures and a full-thickness flap design, was suitable for encouraging stable and successful soft-tissue healing.

### 4.2. Observations on Healing

In this instance, the PI procedure produced great results: the bone defect healed completely in 4 months. Given the existence of a vestibular bone fenestration—which usually presents difficulties because of its position and the thin cortical bone in this region—this quick and efficient healing is very remarkable. CBCT images verifying the preservation of the alveolar ridge proportions highlight the technique’s efficacious prevention of bone resorption.

The PI technique’s ability to preserve the integrity of both hard and soft tissues is further bolstered by the lack of volumetric loss in the soft tissues. This is crucial when placing dental implants because stable soft tissue and enough bone volume are necessary for the long-term durability and aesthetics of the implants.

The comparatively small sample size of this study is a significant restriction that could affect how broadly the findings can be applied. Even if the statistics are encouraging, it is important to recognize that bigger clinical trials are necessary to establish the reproducibility of these findings. Future research with larger sample sizes will be able to draw stronger conclusions and better detect possible differences across various patient populations. Larger-scale study is therefore essential to confirm the effects that have been seen and improve the generalizability of our findings in various therapeutic contexts.

### 4.3. Comparative Analysis with Other Techniques

Guided bone regeneration (GBR) is a well-established technology that has been investigated in the management of vestibular bone fenestration. In order to create a space for bone regeneration and encourage volumetric increase, GBR uses barrier membranes to prevent soft-tissue invasion of the bone defect [21]. Although GBR has shown promise in situations involving significant bone augmentation, it frequently calls for the use of extra graft materials and prolonged healing times, which can raise costs and complicate procedures [13]. The Periosteal Inhibition (PI) technique, on the other hand, provides a less complicated method that uses an externally placed PTFE membrane to prevent osteoclastic activity at the cortical bone surface. This preserves the original bone volume without the need for biomaterials or transplants. Our case shows that the PI approach can produce adequate results in terms of preserving soft tissue and bone in a shorter amount of time, making it a potentially good substitute for instances with mild to moderate abnormalities. To thoroughly assess the benefits and drawbacks of the PI approach in comparison to GBR, more comparative research is necessary, particularly when dealing with patients who have different levels of bone loss and anatomical differences.

## 5. Conclusions

The results of this case study highlight the Periosteal Inhibition (PI) technique’s major benefits for treating alveolar bone abnormalities. The vestibular bone fenestration is a difficult process, but the PI method worked well and had few side effects. It is crucial, however, to understand that a patient’s unique circumstances, including oral hygiene, general health, and surgical skill, are crucial to the outcome of these operations. Even in cases where early healing appears to be progressing nicely, clinicians need to be on the lookout for potential consequences such as infection, membrane exposure, and soft-tissue dehiscence.

The encouraging results shown here imply that the PI approach is a potential treatment option for difficult bone deformities, even if more investigation is required to validate it in a wider range of clinical circumstances. The PI approach was initially created for intact cortical bone without the use of biomaterials, and it has shown promise in enhancing clinical result prediction. By demonstrating that the PI technique can accomplish the full repair of bone defects even in the presence of cortical irregularities like fenestrations, this work expands the application of the PI technique.

The PI technique’s potential versatility is highlighted by its dual action of maintaining existing bone and stimulating the development of new bone through tissue compartmentalization assisted by the PTFE membrane. Future studies could examine its application in the management of bone dehiscence, hence increasing the novel approach’s clinical applicability.

In conclusion, the PI approach is a noteworthy development in bone surgery that provides a reliable and efficient means of managing bone abnormalities. It may also have wider uses in the field of dental surgery.

## Data Availability

All data generated or analysed during this study are included in this published article.
